# Paratuberculosis in small ruminants in the Sudan: prevalence and risk factors

**DOI:** 10.1186/s12917-025-04920-8

**Published:** 2025-07-29

**Authors:** Sanaa M. Idris, Wisal A. Elmagzoub, Julius B. Okuni, Lonzy Ojok, Mohamed E. Mukhtar, Enass M. Abdalla, Sulieman M. El Sanousi, Ahmad Amanzada, Uwe Truyen, Ahmed Abd El Wahed, ElSagad Eltayeb, Kamal H. Eltom, Ahmed A. Gameel

**Affiliations:** 1 Department of Animal Health and Safety of Animal Products, Institute for Studies and Promotion of Animal Exports, University of Khartoum, Khartoum North, 13314 Shambat Sudan; 2 Department of Pathology, Faculty of Veterinary Medicine, University of Khartoum, Khartoum North, 13314 Shambat Sudan; 3 Institute of Animal Hygiene and Veterinary Public Health, Faculty of Veterinary Medicine, University of Leipzig, An den Tierkliniken 1, D-04103 Leipzig, Germany; 4 Department of Biology and Biotechnology, College of Applied and Industrial Sciences, University of Bahri, Khartoum North, Sudan; 5 College of Veterinary Medicine, Animal Resources and Biosecurity (COVAB), Makerere University, P. O. Box 7062, Kampala, Uganda; 6 Department of Pathology, Faculty of Medicine, Gulu University, P.O.Box 166, Gulu, Uganda; 7 Department of Agricultural Extension and Rural Development, Faculty of Agriculture, University of Khartoum, Khartoum North, 13314 Shambat Sudan; 8 Department of Microbiology, Faculty of Veterinary Medicine, University of Khartoum, Khartoum North, 13314 Shamabt Sudan; 9 Department of Gastroenterology and Gastrointestinal Oncology, University Medical Centre Göttingen, Robert-Koch-Straße 40, D-37075 Göttingen, Germany; 10 Faculty of Medicine, Al Neelain University/ Ibn Sina Specialised Hospital, Street 17 -21 Alamarat, Khartoum, 12217 Sudan

**Keywords:** Paratuberculosis, Small ruminants, *Mycobacterium avium* subsp. *Paratuberculosis*, Recombinase aided amplification

## Abstract

**Background:**

Paratuberculosis (PTB), caused by *Mycobacterium avium* subsp. *paratuberculosis* (MAP), is a contagious and chronic enteric disease of ruminants and many non-ruminants leading to emaciation and death of the animal. PTB is poorly investigated in sheep and goats in Sudan, where these animals contribute significantly to food security and poverty alleviation as sources of income. They also play an important role in the national economy through animal exports. The objectives of this study were to investigate the prevalence of PTB and associated risk factors in small ruminants. Blood and faecal samples were collected from 818 sheep and goats aged > 1 year in 111 flocks distributed over five states (Blue Nile, West Kordofan, Khartoum, the Gezira and White Nile) of the country from November 2020 to October 2022. Serum samples were tested by an enzyme-linked immunosorbent assay (ELISA) for detection of MAP antibodies and the faecal samples were tested for MAP DNA using a recombinase aided amplification (RAA) assay.

**Results:**

The overall true animal-level prevalence of PTB was 10.7% by RAA (4.2% in sheep, 6.5% in goats) and 1.7% by ELISA (0.7% in sheep, 1.0% in goats). At the flock level, prevalence was 41.8% by RAA and 8.5% by ELISA. While no significant associations were found between animal-level factors and PTB, several flock-level factors including breed homogeneity, source of new animals, management system, animal movement, separation of sick animals, and flock history of PTB were significantly associated with MAP detection (*P* < 0.05).

**Conclusions:**

The high flock-level prevalence of MAP DNA indicates a potential risk for environmental dissemination, especially under open grazing systems. Despite the relatively low seroprevalence, molecular detection suggests subclinical infection may be underdiagnosed. These findings highlight the importance of using combined diagnostic methods for effective PTB surveillance and control. Improved flock management practices are recommended to reduce MAP transmission and environmental contamination.

**Supplementary Information:**

The online version contains supplementary material available at 10.1186/s12917-025-04920-8.

## Background

Paratuberculosis (PTB) or Johne’s disease is a chronic enteritis affecting mainly domestic and wild ruminants as well as non-ruminant animals [1–3]. It is caused by *Mycobacterium avium* subsp. *paratuberculosis* (MAP) and is of worldwide distribution. Beyond its economic implications in livestock, MAP has raised public health concerns due to its suggested association with certain human diseases [4–7]. Although PTB is widely studied in cattle, its occurrence in small ruminants such as sheep and goats is less well documented. However, it has been reported in small ruminants in Canada [8], Spain [9], USA [10], Germany [11], Poland [12], Italy [13, 14], Latin American and Caribbean countries [15], Saudi Arabia [16], Iran [17], Indies [18], South Korea [19], Tanzania [20]. Nevertheless, the seroprevalence of the PTB in small ruminants was very low at the animal level in the reporting countries.

Infection occurs primarily through the ingestion of MAP contaminated feed or water. The bacteria penetrate the ileal wall and are then taken up by macrophages, where they persist and multiply. This leads to chronic intestinal inflammation and granulomatous lesions [21, 22]. The resulting thickened intestinal wall leads to malabsorption, affects the gut microbiome, and impairs intestinal metabolism, resulting in nutrient depletion, protein loss and muscle wasting [23]. However, in small ruminants, clinical signs are often subtle or absent. Sheep may pass soft faeces and diarrhea occurs in about 20% of infected animals at advanced stages of the disease, while goats may show weight loss and reduced productivity [24, 25]. Importantly, subclinically infected animals greatly contribute to dissemination of MAP in the environment and spread of the disease [26] and diagnostic techniques with high sensitivity and specificity are required. Therefore, for reliable and realistic epidemiological data on PTB and for a better understanding of the dynamic of MAP transmissions between flocks, especially with limitations of diagnostic tests, combination of more than one diagnostic test is likely to give more definite results [27].

Many risk factors of MAP infection at both animal and flock levels have been identified by several studies [28–35]. These factors include age, sex, species, breed, poor biosecurity, introduction of new animals without quarantine, manure management practices and animal density.

In the Sudan sheep and goats have a vital socioeconomic role in the Sudanese community [24]; they contribute significantly to food security and alleviation of poverty and serve as national income through animal exports [36]. This role would be greatly reduced by chronic diseases such as PTB. Despite its potential impact, research on PTB in small ruminants in the Sudan is scarce [37–40], and no prevalence data were available. Without such data, it is difficult to design effective prevention and control strategies for the disease.

Therefore, the present study was undertaken to estimate the prevalence of PTB and identify associated risk factors in sheep and goats across five states of the Sudan.

## Methods

### Study area

**Fig. 1:**
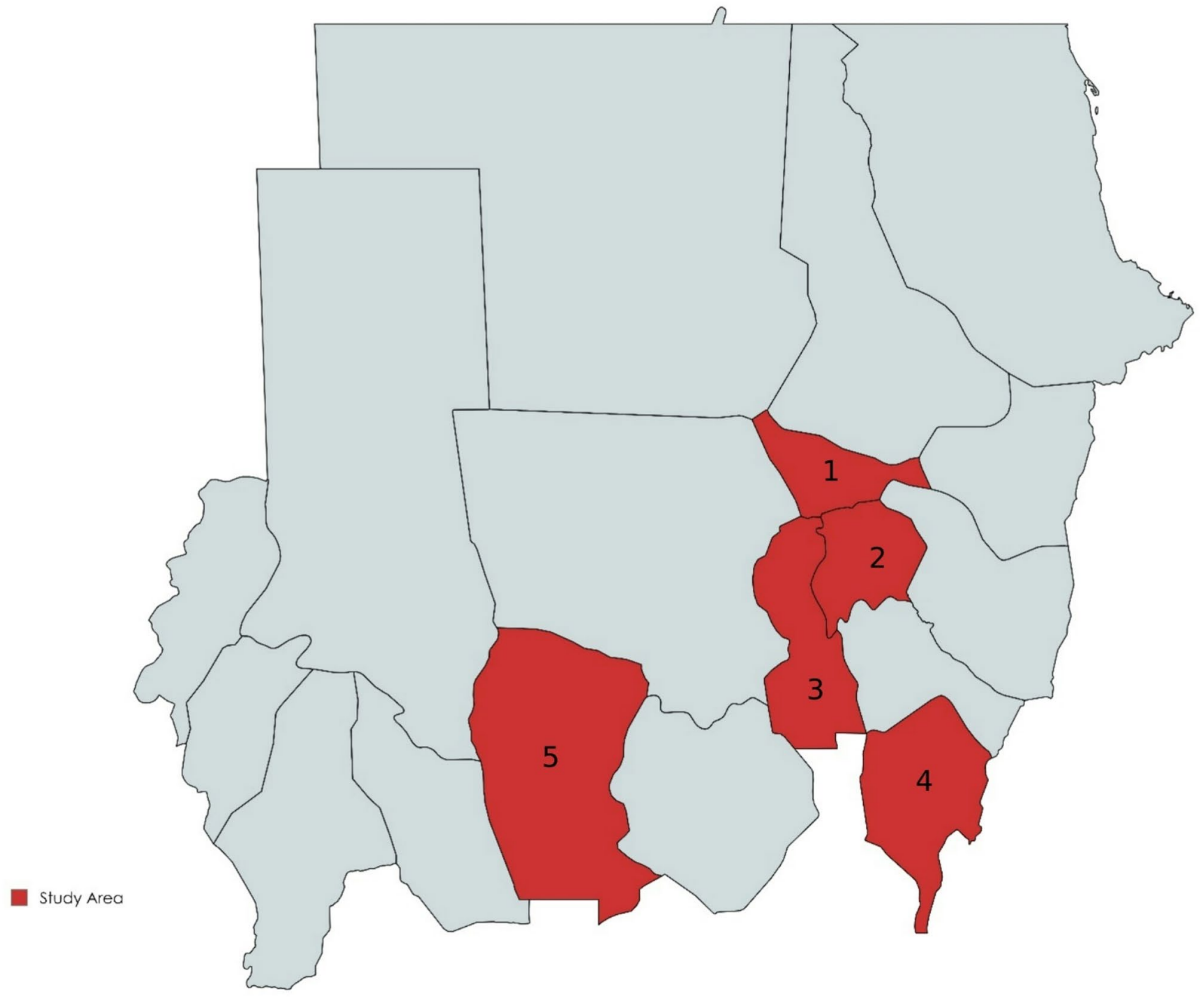
Map of the Sudan showing the states (in red) selected for PTB survey in small ruminants. The map was created from https://mapchart.net/world.htm

Based on the large populations of small ruminants, ecological diversity, and variations in animal breeds (ecotype breeds), five States were selected for this study: Blue Nile, West Kordofan, Khartoum, the Gezira, and White Nile (Fig. 1).

### Collection of samples

#### Selection of the flocks

In the Sudan, small ruminants are predominantly raised under an extensive and semi-extensive production systems [36]. Flocks belonging to different owners often share the same grazing area, and therefore in this study, such shared-grazing flocks were considered a single flock.

#### Sample size at flock-level

The number of such flocks in the same area varies from day to another and the same area can be grazed by different flocks the next day, while the number and sizes of these areas were not fixed. Therefore, the grazing areas were selected based on accessibility as determined by the local guide of veterinary authorities and geographic location representing administrative divisions within the state.

A total of 111 flocks across the 5 states in the 3 regions were included in the study: 37 flocks in the Blue Nile, 22 flocks in West Kordofan, and 52 flocks in the central States (35 Khartoum, 10 the Gezira, and 7 White Nile).

#### Sample size at animal-level

The animal-level sample size was calculated using the Cochran’s formula (Cochran, 1977):

Sample Size = Z² × p × (1 – p) / C².

A Z-value of 1.645 was used, corresponding to a 90% confidence level. The expected prevalence (p) was set at 50%, following Cochran’s recommendation for use in the absence of reliable prior data. This conservative estimate maximizes the required sample size, ensuring sufficient statistical power.

A 5% margin of error (C) was selected to provide precise prevalence estimates. The resulting required sample size was 270 animals for three States (Blue Nile, West Kordofan), and for the cluster of three central States (Khartoum, the Gezira, and White Nile).

Accordingly, a total of 818 fecal and blood samples were collected using stratified random sampling from the selected regions between November 2020 and October 2022. Stratification was based on geographical location, accessibility, flock size, and species (sheep or goats). Within each selected flock, animals were selected randomly for sampling proportional to the flock size, considering the sex as much as possible to reflect the flock structure. Equal numbers of sheep and goats (both sexes and of ages more than 1 year) were targeted, when both species were available for sampling. Samples were collected after obtaining consent of animal owners and permission from the federal and local veterinary authorities, and the ethical approval was made by faculty of Veterinary Medicine, University of Khartoum.

About 4 ml of blood sample was withdrawn from each animal after taking all necessary precautions by jugular venipuncture into plain tubes. About 10 faecal pellets were collected directly from the rectum and put into screw- capped plastic vials. The samples were transferred into a mobile refrigerator to the laboratory at the University of Khartoum. The serum was separated by centrifugation and all samples were stored at – 20 °C until needed.

#### Detection of MAP antibodies

Anti-MAP antibodies in collected sera were detected using a commercial indirect ELISA kit (IDEXX Paratuberculosis screening, IDEXX Laboratories, Inc., Westbrook, USA; Cat. No: P07130-5), according to the manufacturer’s instructions. Briefly, sera and controls were incubated in diluent buffer (1:20) containing *Mycobacterium phlei* for 45 min for removing the cross-reacting antibodies of other mycobacteria. Then 100 µl of the diluted sample was loaded into the 96-well microtiter plate and incubated at room temperature (RT) for 45 min. The plate was washed using the diluted wash buffer (1:20), after which a diluted conjugate (1:100) was added to each well and incubated at RT for 30 min. The plate was washed for a second time before adding 100 µl of Tetramethylbenzidine (TMB) substrate then the plate was covered by aluminum foil at RT for 10 min, followed by addition of 100 µl of stop solution to stop the reaction. The optical density (OD) of the palate was measured at 450 nm using a plate reader (BioChek BV, Reeuwijk, The Netherlands). Positive and negative results were determined according to the manufacturer’s instruction.

#### Detection of MAP DNA

The extraction and detection of MAP DNA was done on the Mobile Suitcase Laboratory developed by Abd El Wahed et al. [41].

#### DNA extraction

The DNA was extracted from feacal samples as follows: about 3 g of faecal pellets were used to make faecal suspension by immersing in 4 ml distilled water. Then the faecal suspension was mixed vigorously and let the suspension for 5 min at RT to settle. Then the upper layer was taken and centrifuged. The sediment was used for DNA extraction according to Hansen et al. [42]. Briefly, 500 µl of lysis buffer were added to about 100 mg of faecal sediment in Precelly’s SK 38 tube (Bertin Corp., Rockville, MD, USA), vortexed for 1 min, 60 µl of well homogenized magnetic beads were added to the sample and vortexed for 10 s. The samples were incubated in a heat block at 95 °C for 15 min with vortexing every 2 min. After a short spin, the sample tubes were placed on magnetic rack for 2 min, 10 µl of clear fluid were transferred to a 0.5 ml tube containing 40 µl of molecular biology grade water. The diluted sample was used as a DNA template for the RAA assay.

#### Recombinase aided amplification (RAA) assay

The isothermal technology was used for detecting MAP DNA in the DNA extracted from faecal samples according to Hansen et al. [43]. An oligo mix was prepared from 2.1 µl of 10 µM of both forward, FP1: 5-CGTGGACGCCGGTAAGGCCGACCATTACTGCATGG-3 and revers RP2: 5- CGCCGCAATCAACTCCAGCAGCGCGGCCTC-3 primers, 0.6 µl of the 10 µM of the exo-probe 5-ACGCCGGTAAGGCCGACCATTACTGCATGGTQTFTAACGACGACGCGCA-3 and 8.2 µl molecular grade water. The oligo mix was added to 29.5 µl of rehydration buffer and 5 µl DNA sample into the freeze-dried reaction pellet of the RAA kit (QT Biotech Co., Ltd. Wuxi China; REF. F00R01A). In the lid of the reaction tube 2.5 µl of magnesium acetate was added before closing the tube. In each run positive and negative controls were included. The short spin, vortexing and short spin again were made for the tubes of RAA reaction before incubated at 42 °C in the tube scanner (Axxin, Fairfield, Australia) for 15 min. The reaction was interrupted by removing the tubes after 3 min, vortexing, short spin and replacing them back into the tube scanner. The results were read on the machine screen in real time.

#### Risk factors associated with PTB prevalence

A structured questionnaire was used to interview animal owners or flockers. The questionnaire was designed to collect information about the animal demography (breed, age, sex, etc.), flock (source of new animals, exposure to wild animals/ other animals, history of PTB, etc.) and flock environment (contamination of water and feed with faeces, etc.).

### Statistical analysis

Data from the ELISA and RAA assays were processed using Microsoft Excel. The true prevalence for the results of was calculated both assays using the EPITOOLS platform [44]. The sensitivity and specificity of each test was adjusted to 90% confidence level according to [45]. The ELISA kit used (IDEXX Paratuberculosis Screening, IDEXX Laboratories, Inc., Westbrook, USA, Cat. No: P07130-5) has 51.4% and 99.3% reported sensitivity and specificity, respectively [46]. In comparison, the RAA assay has a sensitivity of 89.5% and a specificity of 100%, as described by Hansen et al. [43]. The collected data were analyzed by the Statistical Packages for Social Sciences (SPSS) version 20 (IBM SPSS Statistics 20, IBM Armonk, NY, United States), using Chi Square test. The results were presented as percentages and the correlation between the factors was set at p ˂ 0.05.

## Results

A total of 818 small ruminants from 111 flocks across five states in three selected regions, were tested for MAP antibodies and MAP DNA using ELISA and RAA, respectively.

### Seroprevalence of MAP

Antibodies against MAP (seroprevalence of MAP) were detected in 3 goats and 2 sheep out of the 818 serum samples, giving apparent prevalence of 0.6% at the animal level, from which the true seroprevalence was 1.7%. At the flock level, the apparent seroprevalence was 5.1%, while the true seroprevalence was 8.5%. Whitin the individual states, 0.0% seroprevalence was recorded for the Gezira and Blue Nile, while in West Kordofan it was 4.0% followed by Khartoum (3.0%) and the White Nile (1.0%), as shown in Table 1.

**Table 1:** Prevalence of anti-MAP antibodies in serum samples of small ruminants detected by Enzyme-Linked immunosorbent assay (ELISA)

State	No. of tested animal (*n*)	No. of positive Animal (*n*)	Apparent prevalence at animal level (%)	True prevalence at animal level (%)	No. of flocks tested	No. of Positive flocks	Apparent prevalence at flocks (%)	True Prevalence at flock (%)
Khartoum	90	2	2.0	3.0	35	2	5.7	9.0
White Nile	92	1	1.0	1.0	7	1	14.0	20.0
The Gezira	92	0	0.0	0.0	10	0	0.0	0.0
West Kordofan	270	2	1.0	4.0	22	2	9.0	16.0
Blue Nile	274	0	0.0	0.0	37	0	0.0	0.0
	**818**	**5**	**Av. 0.6**	**Av. 1.7**	**111**	**5**	**Av. 5.1**	**Av. 8.5**

### Molecular prevalence of MAP

**Fig. 2:**
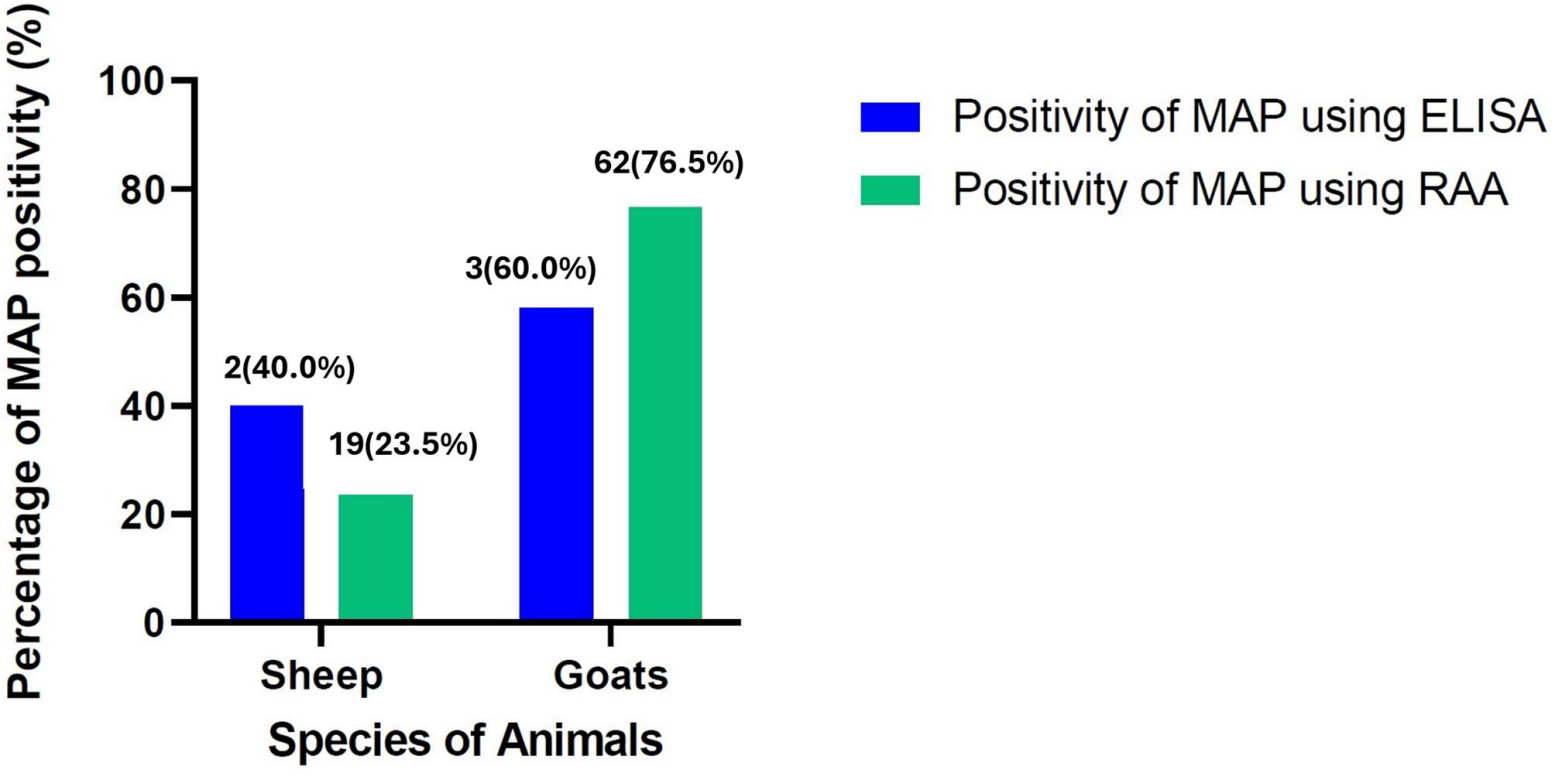
Comparison of mycobacterium avium subsp. paratuberculosis (MAP) positivity in sheep and goats detected by enzyme-linked immunosorbent assay (ELISA) and recombinase aided amplification (RAA)

The DNA of MAP (molecular prevalence of PTB) was detected in 62 goats and 19 sheep out of the 818 faecal samples. The apparent molecular prevalence of PTB at the animal level was 9.1%, while at the flock level it was 37.8%, while the true prevalence was 10.7% (4.2% for sheep and 6.5% for goats) at the animal level and 41.8% at the flock level (Table 2). The positivity to MAP was higher in goats compared with sheep (Fig. 2). Whitin the individual states, MAP molecular prevalence was higher in Khartoum (39%) followed by the Blue Nile (12.0%) and the lowest positivity was detected in West Kordofan (2%) (Table 2).

**Table 2:** Prevalence of MAP DNA in faecal samples of small ruminants detected by the recombinase polymerase amplification assay (RAA)

State	No. of tested (*n*)	No. Positive (*n*)	Apparent prevalence at animal level (%)	True prevalence at animal level (%)	No. of flocks tested	Positive flocks	Apparent prevalence at flock (%)	True Prevalence at flock (%)
Khartoum	90	32	35.0	39.0	35	20	57.1	63.0
White Nile	92	7	7.0	8.0	7	3	42.8	47.0
The Gezira	92	7	7.0	8.0	10	7	70.0	78.0
West Kordofan	270	5	1.0	2.0	22	3	13.6	15.0
Blue Nile	274	30	10.0	12.0	37	16	43.2	48.0
	**818**	**81**	**Av. 9.1**	**Av. 10.7**	**111**	**49**	**Av. 37.8**	**Av. 41.8**

### Risk factors associated with PTB prevalence

Analysis of risk factors associated with the prevalence of PTB showed no significant association (*P* > 0.05) between positivity and animal risk factors (sex, age, and body condition). Also, there was no significant association (*P* > 0.05) between positivity and some flock factors: contact with wild animals and flock size, but significant association (*P* < 0.05) was found between PTB prevalence and breed, source of new animals, raising system, movement out the grazing area, exposure to other domestic animals, separation of sick animals and history of PTB (Table 3).

**Table 3:** Risk factors associated with the prevalence paratuberculosis in sheep and goats in the Sudan

Category	Groups	Prevalence		*P*-value
		Apparent	True	
Flock size	less than 30	13.0% (25/192)	3.1%	0.188
	30–60	10.6% (16/151)	2.0%	
	more than 60	8.4% (40/475)	4.9%	
Age	less than 2 years	9.2% (12/130)	1.5%	0.808
	more than 5 years	11.3% (18/160)	2.2%	
	2–5 years	9.7% (51/528)	6.2%	
Sex	Male	7.9% (5/63)	0.6%	0.587
	Female	10.1% (76/755)	9.3%	
Breed	Local	7.6% (57/747)	7.0%	0. 000
	Local cross	33.8% (24/71)	2.9%	
Body condition	Poor	3.1% (1/32)	0.1%	0.424
	Fair	10.7% (72/707)	8.8%	
	Good	10.1% (8/79)	1.0%	
Raising System	Nomadic	6.2% (38/611)	4.6%	0.000
	Semi-nomadic	9.4% (11/117)	1.3%	
	Intensive farming	35.6% (32/90)	3.9%	
Source of new animals	Purchasing	2.3% (3/132)	0.4%	0.002
	Breeding	10.4% (57/546)	7.0%	
	Purchasing & breeding	15.0% (21/140)	2.6%	
Movement out the flock	Yes	8.2% (57/696)	7.0%	0. 000
	No	19.7% (24/122)	2.9%	
If yes	Same area	10.7% (22/216)	3.2%	0.198
	Multiple areas	7.3% (35/480)	5.0%	
Exposure to wild animals	Yes	10.8% (51/474)	6.2%	0.335
	No	8.7% (30/344)	4.2%	
Exposure to other animals	Yes	7.5% (53/702)	6.5%	0. 000
	No	24.1% (28/116)	3.4%	
If yes, to which animal species	Goat	20.0% (1/5)	0.1%	0.000
	Sheep	50.0% (2/4)	1.0%	
	Cattle, camel, goats, sheep	2.0% (3/148)	0.4%	
	Cattle, goats, sheep	8.6% (47/545)	6.7%	
History of PTB in the flocks	Yes	7.4% (29/390)	3.5%	0. 024
	No	12.1% (52/428)	6.4%	
Separation of sick animal	Yes	31.3% (5/16)	0.6%	0.004
	No	9.5% (76/802)	9.3%	

## Discussion

Paratuberculosis (PTB), caused by *Mycobacterium avium* subsp. *paratuberculosis* (MAP), poses a growing threat to both animal productivity and public health in the Sudan. Many bacterial diseases affecting small ruminant production have been largely controlled through proper diagnosis, treatment, and vaccinations [47]. Despite early reports in the Sudan [40, 48–50], PTB remains neglected. This could be due to poor awareness about the disease, lack of distinguishing clinical signs, unavailability of highly sensitive diagnostic assays [21, 24, 51], and the difficulty in recognizing cases with latent and sub-clinical disease, especially in open pasture.

Small ruminants are hardy animals that cost little to manage and breed. They play an important socioeconomic role in rural communities contributing effectively to poverty alleviation. They provide meat, milk, and skins, and as well as export animals, especially sheep, contributing considerably to the national economy of the Sudan. These roles would be greatly reduced by chronic diseases, such as PTB; PTB results in significant economic losses due to reduced productivity, weight loss, and increased mortality [24]. Moreover, the possible association of MAP with Crohn’s disease and other human diseases [5, 7, 52, 53], has made PTB a public health concern. MAP has been detected in more than 40% of Sudanese patients with gastrointestinal complaints at a referral hospital by real- time PCR and culture [4]. Although the zoonotic potential of MAP remains under investigation, it its detection in cattle, sheep, goats, camels [35, 39, 54], and wild animals (unpublish data), taking into account that the close human-animal interactions are common, should withdraw more attention to PTB. Therefore, this study was conducted to investigate the prevalence of PTB in small ruminants across five Sudanese states.

The results of this showed a 1.7% (0.7% in sheep and 1.0% in goats) true seroprevalence of PTB at animal level, which is relatively low compared to the prevalence in cattle in the Sudan [35]. This result generally agrees with many studies that reported low prevalence of PTB in small ruminants. In Tunisia, a prevalence of 3.25% was reported in sheep [55]. Similarly, a seroprevalence of 2.3% for sheep and 1.0% for goats was found in Grenada, West Indies [18]. In Serbia, prevalence rates of 3.30% were recorded in sheep [56]. On the other hand, the result of this study differs from those of Elsohaby et al. [16] in Saudi Arabia and Pourmahdi Borujeni et al. [17] in Iran, who found the apparent and true seroprevalence of PTB in sheep were 6.87% and 15.37%, and in goats were 7.07% and 15.86%, respectively.

The PTB prevalence at flock level is important for knowing the disease distribution. The PTB true seroprevalence at the flock level in this study was 8.5%, which is low compared with seroprevalence of 18.9% and 66% reported in cattle in the Sudan by Elmagzoub et al. (2020) [35] and Mohammed et al. [57], respectively. Also, low prevalence of 4.2%, and 3.9% was reported in goats in Mexico by Martínez-Herrera et al. [58] and Callejas-García [59], respectively, and 3% prevalence in each of South Africa [13] and southern Italy [60]. But, the present results are relatively higher than the 0.82% prevalence reported in Chile [61], 1.4% in Missouri, USA [10] and 0.8% in South Korea [19] in goats. Comparatively higher rates were also reported: 10.9% in sheep and goats in Tanzania [20] and 16.8% in goats in Brazil [62]. On the other hand, very high seroprevalence has been reported in some countries, 83.0% was reported in goats in Grenada [18], and a similar rate of 83% was found in Ontario, Canada [63]. In Central Italy, seroprevalence in sheep reached 73.7% [64], while in Southern Italy, herd-level seroprevalence reached 71.8% in sheep and 60.8% in goat farms [14]. A prevalence of 65% was reported in sheep in Germany [11], followed by 63.5% in goats in North Gujarat, India [65]. In Poland, 42% of goats were seropositive [12]. The low seroprevalence observed in this study may be influenced by the commercial ELISA kit used, which includes *M. phlei* absorption as a standard step to improve the specificity of the assay by minimizing cross-reactivity with antibodies against other mycobacteria. But, this process can reduce the sensitivity of the ELISA, potentially leading to lower detection rates [66, 67]. Therefore, careful standardization and validation of the absorption step are crucial to achieving an optimal balance between sensitivity and specificity. Moreover, indigenous ELISA kit was found superior in the detection of PTB in sheep and goats in India [68]. However, the detection of MAP-specific antibodies is highly dependent on the stage of infection. In the early stages of the disease, the humoral immune response is often weak and undetectable, leading to false negative results in serological tests. As the disease progresses to a clinical phase, antibody production increases, leading to increased detection by serological assays [69, 70].

On the other hand, in the present study, the true prevalence of PTB was higher when RAA was used compared to ELISA method (10.7% vs. 1.7% at the individual animal level and 41.8% vs. 8.5% at the flock level). The high shedding of MAP in the faeces compared with the presence of MAP antibodies in the serum confirms that more MAP positivity can be obtained when its DNA is targeted rather than its antibodies [16, 39]. The isothermal technique and ELISA are used for detecting MAP DNA and MAP antibodies, respectively, and the isothermal assay has 100% specificity and 89.5% sensitivity [43]. However, for screening PTB in sheep and goats, it is recommended to use more than one test with high sensitivity and specificity. The prevalence of PTB in goats was found to be higher than in sheep. Similar results were reported by Stewart et al. [71] who found that sheep are more resistant to MAP infection than goats. This could be related to the animal species; differences in host response to MAP infection between animal species were detected [72]. Another possible explanation for the present result is that sheep and goats were infected by different strains of MAP, the virulence factors of which could make difference in host- pathogen interactions.

Generally, it can be observed that the molecular prevalence of PTB in West Kordofan was lower (2.0%) compared with Khartoum (39.0%), Blue Nile (12.0%), White Nile (8.0%) and the Gezira (8.0%) States. These variations might be partly attributed to the environmental conditions and/or the type of soil. Clay soils in the central regions and the Blue Nile tend to form lumps or masses with vegetations and faecal matter and make picking of infection more possible compared to sandy soils, as in West Kordofan.

The high prevalence of PTB in many developed countries, such as Germany [11], Italy [64] and Canada [63], compared with the low prevalence in the Sudan, might be due to the highly endemic nature of PTB in these countries, especially in dairy cattle [73]. Also, the wet temperate environment in many countries is conducive to MAP viability [74]. In this respect, it is strongly believed that PTB was first introduced to the Sudan through the importation of foreign European cattle breeds (mainly Friesian) for developing the dairy industry and upgrading the local breeds (Babiker Abbas, personal communication).

In general, the prevalence rates of MAP reported here at the animal and flock levels may be considered low compared with published [35, 57] and unpublished data for cattle in Sudan. This might be attributed to the pastoral system of grazing which minimizes stocking and crowding of animals. Support for this comes from the present finding; 39.0% prevalence of PTB was obtained in Khartoum State, where intensive animal production systems are more established than in other states.

Prevalence and spread of PTB is affected by several factors that have been identified as potential risk factors [30, 31, 75]. It was observed that sex and age of the animal had no significant effect (*p* > 0.05) on MAP prevalence in this study. The same findings were reported by Selim et al. [76], who found no significant effect of age in spreading MAP infection among diarrhoeic sheep. However, other studies found that the total of older goats that tested positive for MAP infection was higher than that of younger ones [77]. Additionally, the young animals and purebred were highly susceptible to MAP infection [28]. These differences in the findings might be attributed to the differences in the sample size of each age group, the age range, or the use of different tests (serological tests versus molecular tests) to detect infected animals. Results of this study showed that introduction of new animals into the flock, through purchase, could be a risk factor for spreading of MAP infection (*p* < 0.05). However, opposing findings were reported by Barrett et al. [75] and Sibley and Orpin [78]. The animal owners in the Sudan prefer breeding for flock growth rather than purchasing. It is important to keep clean animals for purpose of breeding and as safeguard against infection. Carrier animals, whether purchased or bred in the farm, can shed MAP in faeces, milk, colostrum or through the placenta. Furthermore, contact or co- grazing with other animals had significant effect (*p* < 0.05) on spreading of MAP infection. These findings support previous studies [28, 34, 79] and disagree with others [76]. Therefore, the present study suggests that good management practices including separating offspring from dams and the continuous removal of manure from the grazing area would have a significant effect on reducing PTB infection.

## Conclusions

This study represents the first investigation of paratuberculosis (PTB) prevalence in small ruminants in the Sudan and provides valuable insights into the epidemiology of MAP in some regions of the country. The discrepancy between serological and molecular detection rates highlights the limitations of relying on a single diagnostic test, thus emphasizing the need for a combination of diagnostic methods for effective disease surveillance, as subclinical or early-stage infections can be detected by molecular techniques while being missed by serological tests. While this study did not directly evaluate management practices, the findings emphasize the potential importance of effective flock health management including biosecurity measures, appropriate grazing practices, and early identification of infected animals in reducing MAP transmission and environmental contamination. Future research assessing the impact of such measures would be valuable.

## Electronic supplementary material

Below is the link to the electronic supplementary material.

Supplementary Material 1

Supplementary Material 2

Supplementary Material 3

Supplementary Material 4

## Data Availability

All data are available in the manuscript text and in the supplementary files.

## Abbreviations

PTB: Paratuberculosis
MAP: Mycobacterium avium subsp. paratuberculosis
ELISA: Enzyme-linked immunosorbent assay
RAA: Recombinase aided amplification
TMB: Tetramethylbenzidine
OD: Optical density
