# Supplementary material for: Paratuberculosis in small ruminants in the Sudan: prevalence and risk factors
Source: BMC Vet Res. 2025 Jul 29;21:496. doi: 10.1186/s12917-025-04920-8 (PMC12305951; doi:10.1186/s12917-025-04920-8)
Supplement: Supplementary file 4 — Supplementary Material 4 [file 12917_2025_4920_MOESM4_ESM.docx]

**Questionnaire about paratuberculosis in small ruminants. Owner`s name**: **Date**:

**Location specific name**: **Compound**:

**Animal type**:

Goat
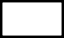
 Sheep

**Flock size**:

Small: <30
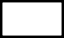
 Medium: 30 – 60
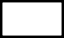
 Large >

**Age**:

2 yrs.
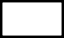
 2-5 yrs.
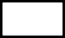
 >5 yrs.

**Sex**:

Male
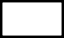
 Female

**Breed**:

Local
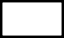
 Local cross

**Body condition**:

Poor
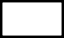
 Fair
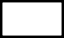
 Good

**Source of food and water supply (Raising system):**

Nomadic
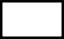
 Semi-nomadic
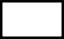
 Intensive farming

**Colostrum and milk feeding:**

always
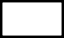
 sometimes
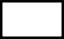
 none

**Source of new animals:**

Purchasing
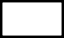
 Breeding Purchasing & breeding

**Manure management:**

Traditional
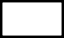
 Modern

**Movement out the flock:**

Yes
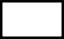
 No

If yes,

Same area
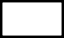
 Multiple areas

**Exposure to wild animals:**

Yes
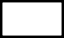
 No

**Exposure to other animals:**

Yes
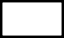
 No

**If yes- type of animals**

Cattle
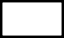
 Camel
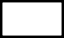
 Goats
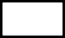
 Sheep
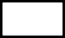
 None

**Historical evidence of PTB in the flock:**

Yes
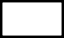
 No

If yes, when

< 2yrs > 2yrs

**Do you separate the sick animal from the flock?**

Yes
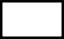
 No

**Is milk area contaminated with manure?**

Yes
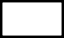
 No

**Is water and feed area contaminated with manure?**

Yes
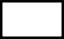
 No
